# Bacterial Communities in Pigmented Biofilms Formed on the Sandstone Bas-Relief Walls of the Bayon Temple, Angkor Thom, Cambodia

**DOI:** 10.1264/jsme2.ME13033e2

**Published:** 2014-07-19

Asako Kusumi^1^, Xianshu Li^1^, Yu Osuga^2^, Arata Kawashima^2^, Ji-Dong Gu^3^, Masao Nasu^4^, and Yoko Katayama^1,2^

^1^United Graduate School of Agricultural Science, Tokyo University of Agriculture and Technology, 3–5–8 Saiwai-cho, Fuchu-shi, Tokyo 183–8509, Japan; ^2^Graduate School of Agriculture, Tokyo University of Agriculture and Technology, 3–5–8 Saiwai-cho, Fuchu-shi, Tokyo 183–8509, Japan; ^3^School of Biological Sciences, The University of Hong Kong, Pokfulam Road, Hong Kong SAR, People’s Republic of China; and ^4^Advanced Pharmaco-science, Graduate School of Pharmaceutical Sciences, Osaka University, 1–6 Yamada-oka, Suita, Osaka 565–0871, Japan

Volume 28, no. 4, Page 422–431, 2013

Page 429, column 2, line 4 from the top, ‘13 mg (g wet weight sample)^−1^’ should read ‘13 μg (g wet weight sample)^−1^’.

Page 429, column 2, line 7 from the top, ‘13 mg (g wet weight sample)^−1^’ should read ‘13 μg (g wet weight sample)^−1^’.

Page 429, column 2, line 14 from the top, ‘0.11 mg (g wet weight sample)^−1^’ should read ‘0.11 μg (g wet weight sample)^−1^’.

Page 429, Table 2, column headings, ‘NO_3_^−^-N (mg [g wet weight sample]^−1^)’ should read ‘NO_3_^−^-N (μg [g wet weight sample]^−1^)’.

